# Approaches for Hybrid Coregistration of Marker-Based and Markerless Coordinates Describing Complex Body/Object Interactions

**DOI:** 10.3390/s23146542

**Published:** 2023-07-20

**Authors:** Hyeonseok Kim, Makoto Miyakoshi, John Rehner Iversen

**Affiliations:** 1 Swartz Center for Computational Neuroscience, Institute for Neural Computation, University of California San Diego, La Jolla, CA 92093, USA; jiversen@ucsd.edu; 2 Division of Child and Adolescent Psychiatry, Cincinnati Children’s Hospital Medical Center, Cincinnati, OH 45229, USA; 3 Department of Psychiatry, University of Cincinnati College of Medicine, Cincinnati, OH 45267, USA; 4 Department of Psychology, Neuroscience & Behaviour, McMaster University, Hamilton, ON L8S 4K1, Canada

**Keywords:** coregistration, image, motion capture, ball object, and juggling

## Abstract

Full-body motion capture is essential for the study of body movement. Video-based, markerless, mocap systems are, in some cases, replacing marker-based systems, but hybrid systems are less explored. We develop methods for coregistration between 2D video and 3D marker positions when precise spatial relationships are not known a priori. We illustrate these methods on three-ball cascade juggling in which it was not possible to use marker-based tracking of the balls, and no tracking of the hands was possible due to occlusion. Using recorded video and motion capture, we aimed to transform 2D ball coordinates into 3D body space as well as recover details of hand motion. We proposed four linear coregistration methods that differ in how they optimize ball-motion constraints during hold and flight phases, using an initial estimate of hand position based on arm and wrist markers. We found that minimizing the error between ball and hand estimate was globally suboptimal, distorting ball flight trajectories. The best-performing method used gravitational constraints to transform vertical coordinates and ball-hold constraints to transform lateral coordinates. This method enabled an accurate description of ball flight as well as a reconstruction of wrist movements. We discuss these findings in the broader context of video/motion capture coregistration.

## 1. Introduction

Motion capture systems have been widely used to track human movements as they provide important scientific data. Out of several technologies that capture body motion, marker-based motion capture has been the standard and most spatiotemporally accurate, though requiring the attachment of physical markers. Markerless video-based motion capture is a promising alternative used in situations where physical contact may not be possible [1,2,3]. It is good for the classification of movements but not yet with the spatial precision of marker systems.

There is a third situation that has had little discussion, which is when a hybrid of the two approaches provides the best performance, such as in ball sports where it is not possible to place active markers on the ball, and passive markers may interfere with the handling and flight of the balls. In such a task, humans interact with physical objects, and thus, both human and object movements must be recorded precisely. While a coarse video-based estimation of human movements has been used in sports such as basketball [4], volleyball [5], soccer [6], and baseball [7], our study of the neural dynamics and biomechanics of juggling needed the precision of an active, high spatiotemporal precision active marker-based system, meaning that the ball motion had to separately be tracked using video, requiring the development of a coregistration system that could be applied to any hybrid motion capture scenario.

Specifically, we apply the hybrid system to the understanding of the motor control of juggling, a new paradigm for computational neuroscience of complex motor skill learning that builds on the simpler tasks that have dominated the field. Its study is possible via the method of Mobile Brain/Body Imaging (MoBI) [8], which uses freely moving participants and wireless EEG to avoid the physical constraints of other stationary brain imaging methods. Three-ball cascade juggling is a basic juggling pattern with three balls that has been extensively studied behaviorally [9,10,11] and which we are proposing as a new model for the study of neural dynamics of motor control during complex skill learning. Juggling involves the cyclic movement of hands and balls; the ball cycle is commonly divided into free-flight and in-hand phases (commonly termed the dwell/hold phase). The ratio between the amount of time the hand holds a ball and the amount of time the hand is empty has been referred to as the dwell ratio, which has been treated as an important parameter for analysis in juggling. A second critical parameter for our neural analysis is the relative location of the ball’s apex to the hands, a key description of juggling stability that is needed to understand the neural control of throws as well as how the visual estimation of ball trajectory influences the motor control of the catch [12,13,14]. Thus, it is imperative to accurately estimate the accurate movement of the hands and balls to define the catch, throw, and apex positions of the ball in the world coordinate space of the hands. In this experiment, the body location is collected in a real-world coordinate system using motion capture equipment, whereas the positions of the balls are captured on video only, and thus, there is no ground truth for the ball position. The true hand position is also unknown, and this necessitates a way to reconstruct it jointly with the unknown ball position. We expect specific use cases that are already active areas of research beyond our application to juggling, such as the study of the relation between a pro tennis player’s serve and ball velocity [15] or between elbow stress and ball trajectory [16]. These are all scenarios in which information about either object kinematics or body kinematics is not sufficient.

In this study, we seek to develop a practical system to integrate commodity video cameras into a marker-based mocap that does not rely on a priori calibration nor on precise knowledge of the camera position and lens characteristics. Such a system could be computationally efficient enough to learn coregistration in real time, which is especially important when the participant is moving relative to the video image plane. In the case of manipulating objects that are alternately held and thrown, two primary constraints are available to be used in coregistration: a positional constraint during holding (ball and hand position coincide) and gravitational dynamics during ball flight. Several additional challenges of motion capture during juggling had to be overcome. Ball position estimation from the video may be inaccurate because of partial occlusion of the ball by the hand, and it was not possible to know the actual hand position because active markers on the hand would be occluded, and thus, hand position was estimated from forearm and wrist markers. Our coregistration method had the secondary benefit of enabling us to estimate the correct hand position, including wrist bending movements that are important for throwing and catching.

Here, we compare multiple methods for the coregistration of video and marker-based motion capture, each differing in their use of hand positions and gravitational constraints. Coregistration methods were evaluated by (1) the difference (error) between the estimated hand position and the estimated ball position during the hold phase and (2) the vertical acceleration of the balls. We chose linear methods for simplicity, assuming that the video plane was spatially linear, but these methods could be easily generalized to use nonlinear scaling methods. The proposed algorithms differed in their use of physical constraints and assumptions to reconstruct the ball trajectory from video, producing trajectories in body space. We provide guidance on the optimal reconstruction of object positions from video that are widely applicable to hybrid video/motion capture scenarios. We expect this discussion to be useful to researchers who used to use marker-based motion capture and vision-based motion capture simultaneously, even in a limited hardware environment.

## 2. Methods

### 2.1. Data Acquisition

**Figure 1:**
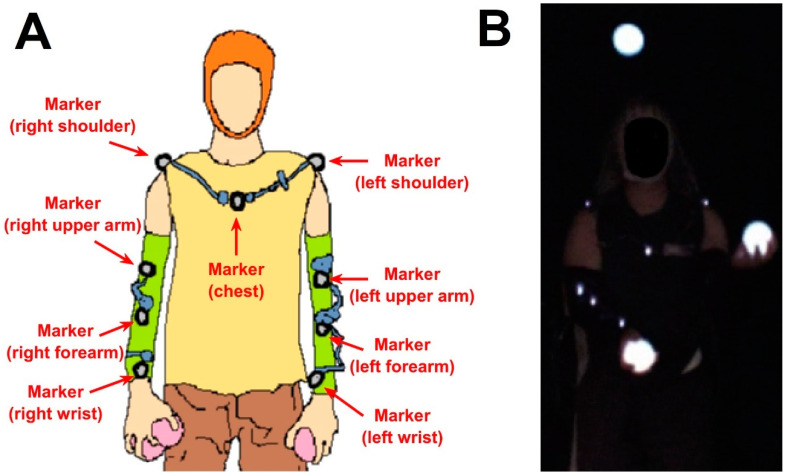
(**A**) Marker positions for body motion capture. (**B**) Video image for ball motion capture.

We recruited healthy subjects who can already juggle. Their age was 28.69 ± 5.66 (mean ± standard deviation). The subjects performed three-ball cascade juggling with internally illuminated balls with a diameter of approximately 7 cm for five 10 min sessions with a 3 min break session between sessions. All participants provided informed consent, and this study was approved by the IRB of UCSD and carried out complying with the Declaration of Helsinki. We used a PhaseSpace motion capture system (PhaseSpace, San Leandro, CA, USA) with a sampling rate of 480 Hz to track nine markers on left/right shoulder, left/right upper arm, left/right forearm, left/right wrist, and chest, as shown in Figure 1A. Each marker was a 1 $\;{\mathrm{mm}}^{2}$ LED emitter mounted on a small circuit board with dimensions 20 mm × 14 mm × 3.2 mm. As balls could not be instrumented with markers, we recorded video of illuminated balls using a Flea2 video camera (Point Grey, BC, Canada) with a frame rate of 60 Hz (Figure 1B). Motion capture system cameras have 3600 × 3600 resolution. The video camera had a resolution of 640 × 480. In addition, participants wore an EEG cap to record EEG data, which was not used for this present study. We used the Lab Streaming Layer (LSL) protocol for temporal synchronization of all recordings [17].

### 2.2. Preprocessing

**Figure 2:**
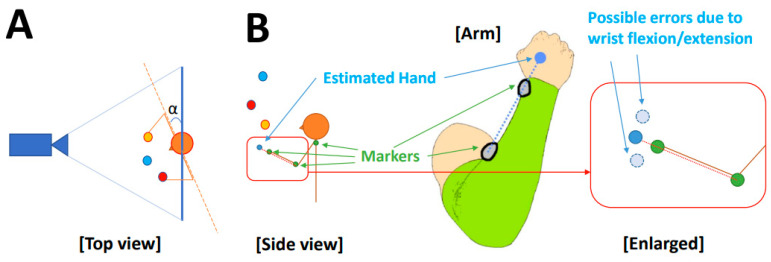
Illustration of the scenario. This illustrates the measurement environment. (**A**) Top view of a performer juggling in front of the camera, but with a possible position offset and angle α between the body plane (defined by shoulder markers; orange dashed line) and camera image plane (thick blue line). (**B**) Side view illustrating hand position estimation by extending a line connecting forearm and wrist markers by 10 cm to estimate the palm center. As shown in the inset of B, the initial hand estimate neglects wrist bending, which our method also aimed to recover.

For motion capture data, juggling trials were first isolated, and all coordinate signals were manually inspected to eliminate abnormal discontinuous points (noise) and interpolate short missing intervals with spline interpolation. Missing intervals that lasted over 0.2 s were not corrected and were discarded in further processing. Then, using the interpolation function interp1() in MATLAB, the motion capture data were downsampled for the purpose of finding the ball coregistration transformation and aligned with the 60 Hz video sample times. Ball positions on a pixel scale were estimated from the video frames by applying an automatic circle-finding algorithm using the circular Hough transformation [18], which is capable of capturing variable circle sizes simultaneously. Video coordinates ran in opposite directions as the motion capture coordinates and were, thus, first inverted. As the recorded video contained only two-dimensional data in a plane approximately parallel to the body plane, it captured only vertical and lateral movements (See Figure 2). The ball depth position was reconstructed by adopting the hand depth position for the hold phase and for the flight phase, linearly interpolating between the depth dimension of the ball position at throw and catch, assuming a constant horizontal ball speed during flight. By construction, this yields real-world scaling, and thus, ball depth was not further considered in coregistration.

To define the body plane, we tracked a rigid body formed by the coordinates of the two shoulder markers and the chest marker. The position and angle of the body plane relative to the video image plane can be used to trigonometrically correct ball coordinates so that they lie in the body plane (Figure 2A). In practice, in this experiment, participants stood on a clear marker, and thus, movement of the body relative to the camera was minimal. The mean and standard deviations for translational movements across subjects were 1.57 ± 0.62 [cm], 0.54 ± 0.23 [cm], and 2.16 ± 0.66 [cm] for lateral, vertical, and depth coordinates, respectively. These shifts, when projected to the video plane, were less than a quarter of the ball’s diameter. The standard deviation of body to video plane angle was less than one degree (0.24 ± 0.13 [degree]) and similarly of no importance. Since all the values were minimal, we neglected the variation in them for further analysis. We also ignored lens distortions and assumed that pixel space was linear. In situations where these assumptions do not hold, these factors could readily be accounted for via a video de-warping transformation followed by simple trigonometric time-varying rotation of ball coordinates into the body plane prior to proceeding with coregistration.

It was not possible to track the hand using active markers due to occlusion of palm-side markers by the ball and invisibility of markers on the back of the hand. While this could be solved in future studies using inertial sensors or placing mocap cameras below the hands, it was a limitation inherent in this data, and thus, recovery of hand position was a secondary aim of our method. We proceeded by estimating the hand position using wrist and forearm markers by extending a line connecting the two markers by 10 cm (the average distance from wrist marker to palm center, as shown in Figure 2B). Our simple estimate of hand position neglects flexions/extensions, which are typically on the order of 5 cm, or about a ball diameter, in the vertical direction, as well as lateral deviations, which are generally smaller, on the order of 2 cm. Thus, most of the remaining variation seen after the coregistration can be reasonably ascribed to wrist movements. The balls and hands are, thus, mutually constraining, which provides a method not only to coregister the balls into the body space but also estimate the true hand position incorporating wrist bending.

To determine the hold and flight phase of the balls, throw and catch events were manually inserted based on inspection of the video and verified by a second observer. The hold phase was defined as an interval from catch to throw, and the flight phase was defined as an interval from throw to catch. As the frame rate was 60 Hz, the time of catch and throw could each be wrong by a maximum of 16.7 ms. We did not interpolate the exact time between frames, which could maximally distort the duration of hold and flight phases by approximately 6.2% (the phase duration was approximately 520 ms, averaged across subjects.). Since misalignment of events could misclassify the phase of motion samples near the throw and catch, we removed 3 samples (~50 ms) at the beginning and end of each phase. This had the side effect of eliminating any frames in which ball occlusion by the hand may have led to ball position estimation errors.

### 2.3. Coregistration Methods

Ball coordinates derived from the video data on the lateral and vertical axes are on a pixel scale, and the aim of coregistration is to match these to the real-world scale. The units can be converted using a linear equation, as we confirmed the validity of linearization in the previous section. Linearity enables us to compare methods with ease and has high generalizability in general.

As there is no a priori best method for reconstructing the ball position from video, we explored the following task-dependent constraints: (1) the ball position during the hold phase should be identical to the hand position; (2) during free flight, the ball is subject to gravitational acceleration of approximately 9.8 [$m/s^{2}$] at our latitude [19]. The first constraint was only approximate, given the need to estimate the hand position discussed above, and thus, a secondary outcome of coregistration is to recover the hand position. Prior to registration, ball and hand coordinate data were segmented into hold phase segments for each hand and flight phase segments for each ball. These were separately concatenated. This was justified by our determination that body movement relative to camera was a negligible source of error, so we could fit the entire recording at once. In situations where body movement was larger, coregistration fitting would be preceded by a transformation of ball coordinates into the 2D plane of the body using continuously time-varying shifts and rotations of the body relative to the camera.

We considered four possible approaches for coregistration, varying in their use of two task constraints (hold vs. flight phases) and whether vertical and lateral axes were scaled uniformly or separately.

1. **Ind**, the naïve approach is to minimize the distance between hand and ball during the hold phase, in a linear least square sense, **Ind**ependently for the vertical and lateral axes.
2. **L**, deriving a **L**ateral scale designed to match the ball and hand positions at the extreme lateral ends of the movement range, which is often approximately near the point of ball catching. Such an approach reasons that scaling fits will be most accurate when using extreme values (defined as the maximal 2.5% of absolute lateral hand coordinates for both right and left hand, as slope errors are greater for points more closely spaced). The mean of the ball position was matched to the mean of the hand position.
3. **V**, deriving a **V**ertical scale such that the vertical acceleration of the ball while in flight is 9.8 [$m/s^{2}$] and applying this same scale to the horizontal axis.
4. **LV**, combining the results obtained from (2) for the lateral axis scaling and (3) for the vertical axis scaling.

Note, Ind and LV (methods 1 and 4) permit different scaling for vertical and horizontal axes, while L and V (methods 2 and 3) use a common scaler for both axes. Satisfying the theoretical acceleration of gravity constraint using ball flight phase is trivial, but there are various ways to try to satisfy the hold phase constraint. Thus, we applied the least square fitting to take advantage of the entire hold-phase information (Ind, method 1).

The performance of each method in matching the two essential constraints was assessed using a ball–hand distance metric as well as quantifying the vertical acceleration of balls during flight. The difference (error) between the estimated hand position and the estimated ball position during the hold phase was calculated as follows:

(1) $$\frac{1}{T}\overset{T}{\underset{t=1}{\sum }}\sqrt{{\left(X_{ball}\left(t\right)-X_{hand}\left(t\right)\right)}^{2}+{\left(Y_{ball}\left(t\right)-Y_{hand}\left(t\right)\right)}^{2}+{\left(Z_{ball}\left(t\right)-Z_{hand}\left(t\right)\right)}^{2}},$$

where T means the number of samples in the hold phase for all the trials, and X(t), Y(t), and $Z\left(t\right)$ represent the lateral, vertical, and depth position of the estimated hand position and ball position, respectively. The error was averaged across subjects for group-level comparison. Note that $Z_{ball}\left(t\right)-Z_{hand}\left(t\right)$ was always zero in all four methods because of the way the Z coordinate was defined by hand coordinates. The vertical acceleration of the balls was computed by applying one-sided finite difference twice using all points of the flight phase cut at both ends. All computation and statistical tests in this present study were performed using MATLAB R2022b (MathWorks, Inc., Natick, MA, USA).

## 3. Results

**Figure 3:**
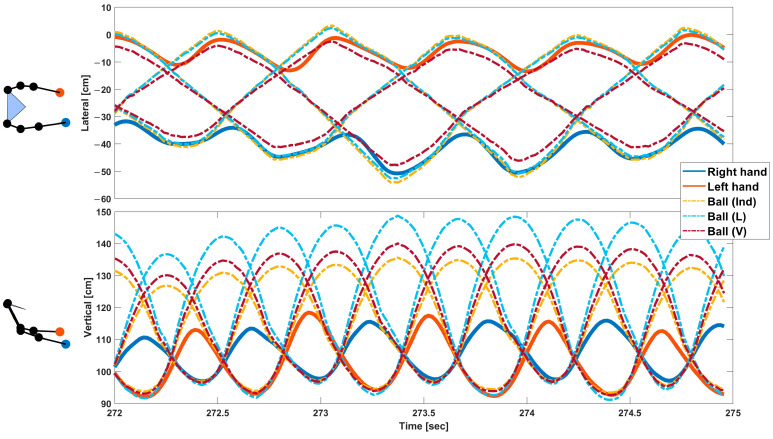
Example of the estimated hand trajectory and coregistered ball trajectory for three hand cycles for a single juggling trial of a representative participant. The plots show the lateral and vertical coordinates, respectively. The thick blue line represents the trajectory of the right hand, and the thick red line represents the trajectory of the left hand. The dashed lines represent the trajectory of the balls coregistered by the proposed methods. The LV method is not here shown separately as it matches the L method in the top panel and the V method in the bottom panel.

As the true trajectory of the ball in real-world coordinates and units is unknown, we reconstructed the trajectories with the four proposed methods from coordinates in the video image plane, enabling us to make practical suggestions of the best approach to reconstructing real-world coordinates from the video. Figure 3 shows an example of the estimated hand trajectory and coregistered ball trajectory during juggling using the proposed methods. Differences between the methods were clearly observed in the lateral registration of the ball and hand during the hold phases and at the ball flight apex in the vertical trajectory.

**Figure 4:**
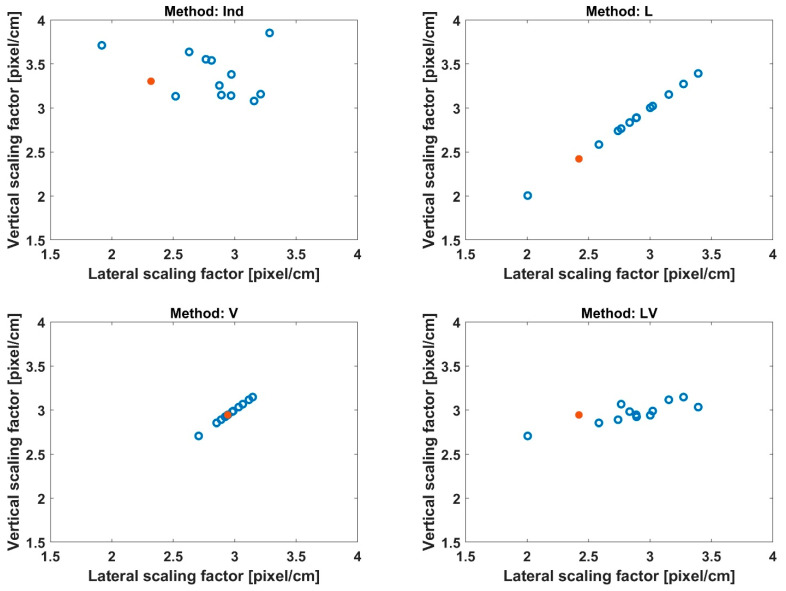
Vertical and lateral coregistration scaling factors obtained from the four methods. Each point corresponds to a subject. The example subject shown in Figure 3 is indicated by a filled red point. As the L and V used a common scale for both axes, all the values lie on the identity line. The Ind and LV revealed that lateral scales were more variable across participants than vertical scales.

Figure 4 shows the vertical and lateral scaling factors obtained from the four methods. The mean of the lateral scales across individuals was 2.79, 2.84, 2.96, and 2.84 [pixel/cm] for the Ind, L, V, and LV, respectively. The mean of the lateral scales across individuals was 3.38, 2.84, 2.96, and 2.96 [pixel/cm] for the Ind, L, V, and LV, respectively. The Ind and LV fits revealed that lateral scales were notably more variable across individuals than vertical scales.

**Figure 5:**
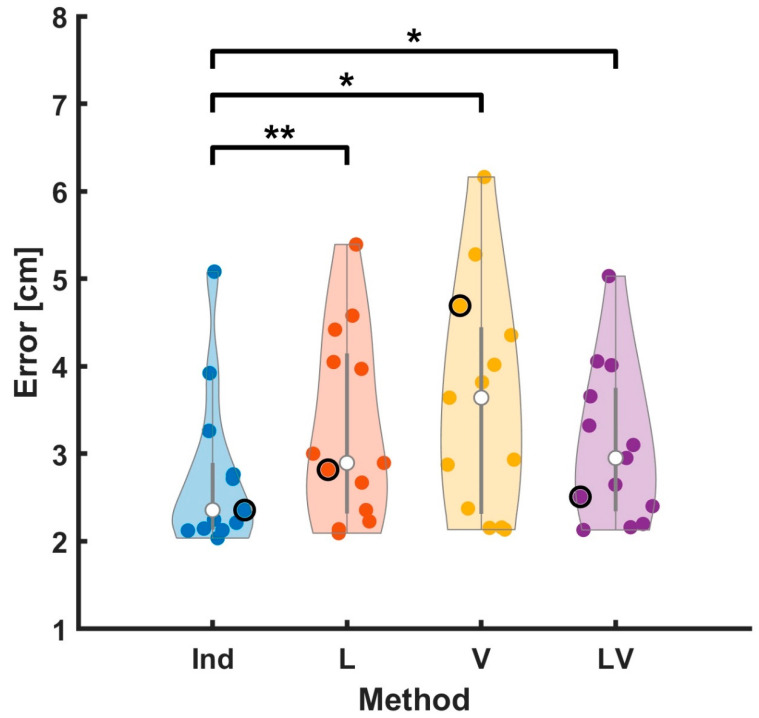
Coregistration distance errors obtained from the four methods. The error was defined as the difference between the coregistered ball position and estimated hand position during the hold phase. Each colored point corresponds to a subject (horizontally jittered for legibility). The white points indicate the median and the gray lines encompass the middle quartiles. The shape of the shaded regions was determined by a kernel density estimate, showing the relative distribution of errors for each method. The example subject shown in Figure 3 is indicated by a black outline. The Ind method showed the lowest error of the methods (t (12) = −3.48, *p* < 0.01 for comparison between the Ind and L; t (12) = −2.88, *p* < 0.05 for comparison between the Ind and V; t (12) = −3.02, *p* < 0.01 for comparison between the Ind and LV). The other comparisons did not show any significant differences. The mean errors across subjects were 2.75, 3.28, 3.58, and 3.09 [cm] for Ind, L, V, and LV, respectively. For comparison, a paired sample *t*-test was performed, and statistical significance was determined using an uncorrected *p*-value. *** *p* < 0.001, ** *p* < 0.01, and * *p* < 0.05.

To compare the performance of the four coregistration methods, we first assessed the position error between the ball and hand during the hold phase. Figure 5 shows the position errors for the four methods for each subject. The mean errors across subjects were 2.75, 3.28, 3.58, and 3.09 [cm], for Ind, L, V, and LV, respectively. The Ind method resulted in the lowest error of the methods and showed a significant difference in all comparisons (t (12) = −3.48, *p* < 0.01 for comparison between the Ind and L; t (12) = −2.88, *p* < 0.05 for comparison between Ind and V; t (12) = −3.02, *p* < 0.01 for comparison between the Ind and LV). The other comparisons did not show any significant differences.

**Figure 6:**
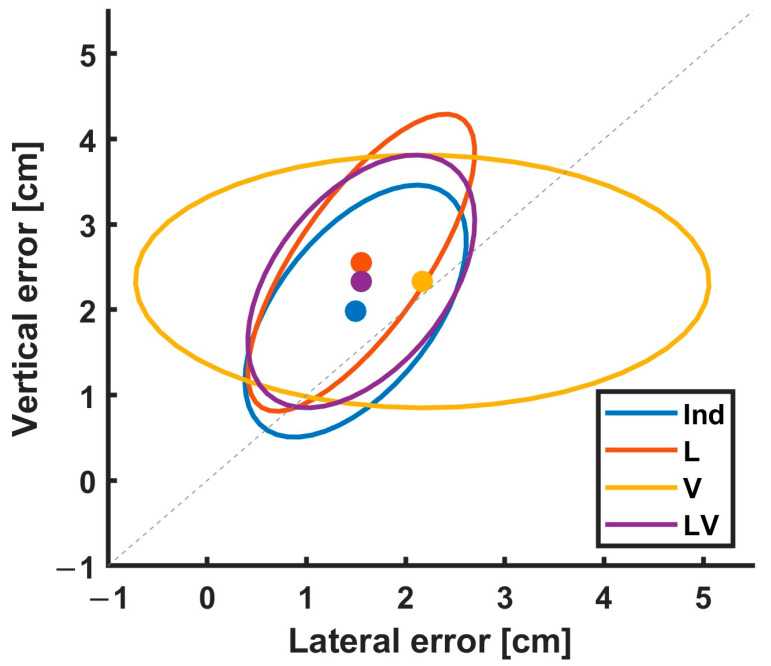
Lateral and vertical error distributions for each method. The ellipse represents a 95% contour of a two-dimensional Gaussian distribution of between-subject variations in error. The shape of the ellipses was determined via fitting two-dimensional Gaussian distribution to the samples on the error space. The ellipse contour is equivalent to the standard deviation of 2. Each dot indicates the mean of the errors across subjects for each method. The grey dotted line is the identity line. The points indicate that the vertical error contributed to the total error more than the lateral error as the mean is above the identity line in all methods, except for V. In addition, while the across-subject variance of V was dominated mainly by lateral error, the across-subject variance of Ind, L, and LV was balanced, indicating that the L method did not preferentially optimize lateral errors.

To establish the relative contribution of errors in the vertical and horizontal axes for each method, we examined the error distribution, as shown in Figure 6. The plot indicates that the vertical error contributed to the total error more than the lateral error as the mean is above the diagonal identity line. In addition, while the across-subject variance of V was dominated mainly by lateral error, the across-subject variance of Ind, L, and LV was more balanced and similar, indicating that the L method did not preferentially optimize lateral errors. In contrast, the V method indicated that the gravitational constraint did poorly at fitting the lateral position. As expected, Ind had the best compromise between bias and variance since it merely found the best fit without incorporating other constraints.

**Figure 7:**
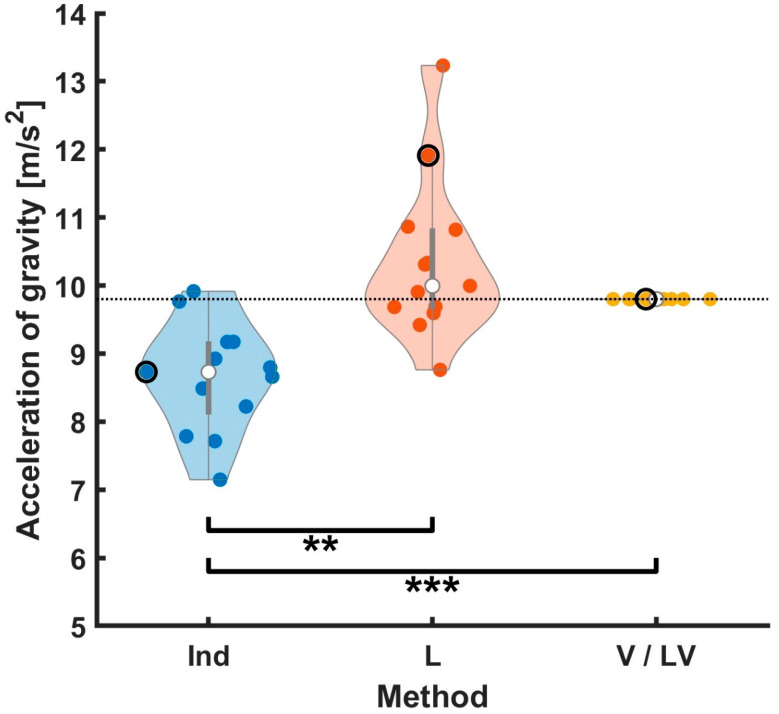
Evaluation of vertical fitting performance by comparing estimates of acceleration of gravity resulting from the methods. Each colored point corresponds to a subject. The white points indicate the median, and both edges of the grey lines indicate the first and third quartiles, respectively. The shape of the shaded regions was determined using a kernel density estimate. The dotted line is located at nominal gravitational acceleration of 9.80 [$m/s^{2}$]. The example subject shown in Figure 3 is indicated by a black outline. The magnitude of gravity estimate using the Ind method was significantly lower than the other methods (t (12) = −3.61, *p* < 0.01 for comparison between the Ind and L; t (12) = −5.20, *p* < 0.001 for comparison between the Ind and V/LV). The L and V methods did not show any significant difference. The mean estimated acceleration of gravity across subjects was 8.65, 10.35, and 9.80 [$m/s^{2}$]. By design, the V and LV methods were constrained to match the theoretical acceleration of gravity. For comparison, a paired sample *t*-test was performed, and statistical significance was determined using an uncorrected *p*-value. *** *p* < 0.001, ** *p* < 0.01, and * *p* < 0.05.

For evaluation of vertical axis coregistration accuracy, we measured the acceleration of gravity from the ball flight trajectories estimated by each method (Figure 7). By construction, vertical scaling in V and LV was determined by fitting the flight trajectories to known gravity, so naturally, these fulfilled the constraint without error. The estimate using the Ind method (8.65 [$m/s^{2}$])was significantly lower than the other methods (t (12) = −3.61, *p* < 0.01 for comparison between the Ind and L; t (12) = −5.202, *p* < 0.001 for comparison between the Ind and V/LV), while the estimate for the L method was higher (10.35 [$m/s^{2}$]) but not significantly, so the L and V methods did not show any significant difference. Clearly, the methods that minimized ball-hand distance during the hold phase failed to properly match gravity during the flight phase.

Taken together, the two evaluation indicators revealed that the Ind method had the lowest error and yet the poorest matching of ball flight to known gravitational acceleration. The V method based on the theoretical acceleration of gravity showed the highest position error during the hold phase, indicating that a uniform scaling of vertical and horizontal coordinates is not optimal.

**Figure 8:**
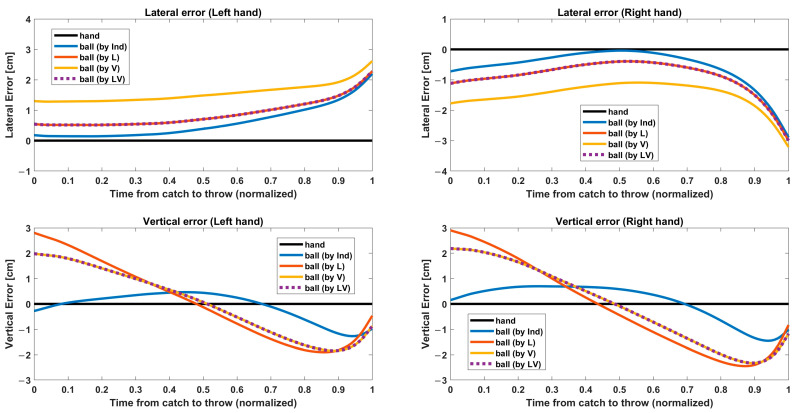
Relative ball trajectories with respect to the estimated hand position in the interval from catch to throw. The interval was temporally normalized, and the normalized trajectories were averaged across subjects. Rows correspond to coordinates (lateral/vertical), and columns correspond to hand (left/right). For vertical position, ball estimate is above hand estimate at catch, dips down before throw, and rises again at throw. For the lateral position, a great error emerged immediately before throw. We observed the trajectory pattern of the V and L was similar, but the Ind had its own pattern, suggesting that the Ind just distorted error trajectory to focus only on minimizing errors. We confirmed that the lowest error of Ind should not be regarded as a good result of coregistration.

Knowing that our target hand position is an estimate, it may not be surprising that merely fitting the ball-to-hand estimate yielded poor global performance. In order to understand this as well as explain the presence of residual position error seen in all coregistration methods, we examined the temporal variation of the ball-to-hand error during the hold phase. Figure 8 shows relative ball trajectories with respect to the estimated hand position. The overall position errors shown in Figure 5 can be regarded as time averages of these curves. As the hold phase had a different length every cycle and across individuals, the interval between catch and throw was temporally normalized, and the normalized trajectories were averaged across subjects. Rows in Figure 8 correspond to the direction (lateral/vertical), and columns correspond to the hand (right/left). The absolute displacements of the ball from the hand estimate are small (<3 cm) but systematic and likely reflect wrist flexion. Indeed, an analysis of these errors allows us to recover a time series for wrist flexion that was not directly measured. For the vertical position, ball estimation is above the hand estimate at the catch, dips down before the throw, and rises again at the throw, consistent with the expected wrist extension and flexion during the catch/throw cycle. The total amplitude of motion, ~5 cm is consistent within the flexural range of motion for the wrist. For the lateral position, the largest error emerged immediately before the throw. This likely reflects a medial rotation of the wrist such that the wrist flexion used to accurately throw the ball across the midline was visible in both vertical and horizontal coordinates. For the lateral coordinates, since we fit left and right hands together, it is expected that left/right symmetry is enforced, explaining the zero bias for lateral ball position relative to left and right hands, although the specific shape of the trajectories was not symmetric, suggesting an average left/right asymmetry in wrist motion. The small bias (~1 cm) around the catch time may be attributed to the use of the most extreme lateral values for fitting in of the L method as it is possible that the wrist marker, which was attached at the wrist of a neoprene body suit, may be systematically shifted at those positions.

## 4. Discussion

### 4.1. Summary of Findings

For the study of body movement, marker-based motion capture systems and markerless motion capture systems have generally been separately used in different situations. However, a hybrid system has important use cases and has been less explored. Here, we developed methods to coregister pixel coordinates extracted from 2D video to 3D real-world positions that do not rely on precise knowledge of the camera and object positions. We developed easy-to-implement methods that could be used in other applications and easily extended to correct for video spatial nonlinearities as well as the movement of the subject relative to the video plane. We applied the methods to a three-ball cascade juggling task where, for the analysis of neural mechanisms of motor control and spatial perception, it was essential for the balls and hands to be spatially co-registered. In the experiment, it was not possible to place active motion-capture markers on the balls due to interference with the handling and flight of the balls, and thus, video recording was used to capture ball position. Secondarily, it was not possible to place motion-capture markers on the back of the hand due to occlusion. The coregistration of the ball-to-hand motion capture data was required to properly characterize spatial dynamics and had the added benefit of enabling us to recover unmeasured wrist movements defining hand movements.

We proposed four methods to combine video and marker-based motion capture and evaluated their ability to correctly coregister balls in real-world space, as well as to learn about unmeasured wrists important for analyzing the fine motor control of juggling. The methods differed in the ways that they utilized key task constraints: gravitational acceleration during the free flight of the balls and identical position of hand and ball during the hold phase. We evaluated the performance of these models using the position error between the reconstructed ball position and the estimated hand position during the hold phase and on their recovery of the correct vertical acceleration of the balls during flight.

The Ind method, which naïvely found the best linear fit between the ball and the hand during the hold phase, naturally resulted in the lowest ball-to-hand position errors but did not properly reconstruct free-flight acceleration, indicating that accounting for observed position differences between the ball and our hand estimate (which was merely an abstraction of a hand without wrist movement) is essential (see Section 4.2, below). In contrast, the V method, which optimized free-flight trajectories, offered, by design, the best physical realism of ball trajectories but resulted in large across-subject variance in lateral position errors. Unlike the V method, the L method showed a balanced across-subject variance, and its vertical variance was similar to the V method. However, the vertical acceleration estimate for the L method was not 9.8 [$m/s^{2}$] and had different values for individuals, ranging from approximately 9 to 13 [$m/s^{2}$] (Figure 7). For the LV method, we observed a balanced across-subject variance and the acceleration estimate of 9.8 [$m/s^{2}$]. In short, only using positional constraints yields a poor estimate of gravitational acceleration.

### 4.2. Recovery of Hand Movement Details

**Figure 9:**
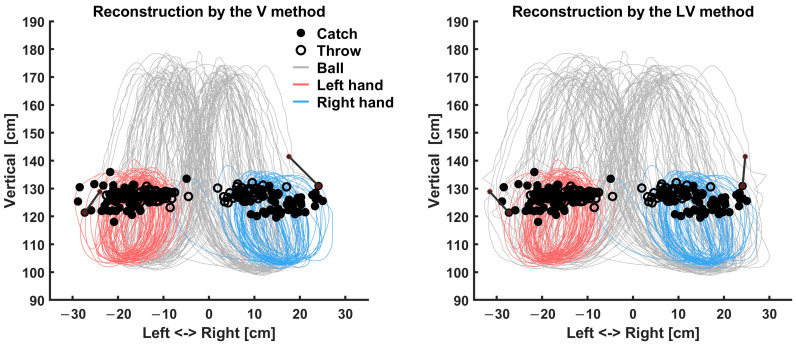
Comparison between the results by the V method (left panel) and LV method (right panel). Data are shown from 50 juggling cycles for one subject. The dark gray lines represent the reconstructed ball trajectory, while the red and blue lines represent the hand position estimates. Filled circles indicate the hand location at catch for each cycle; open circles indicate the throw location. To illustrate position deviations, a dark black line connects the hand position at catch with the reconstructed ball location for the most laterally extreme catches. Both methods resulted in the same vertical offset, but the V method yielded a larger lateral offset, meaning the balls would have to have been caught with a physically impossible movement of the wrist. The LV method shows wrist bends that are physically possible.

One reason for the poor performance of the naïve Ind solution is that the hand position used in the coregistration was an imperfect estimate based on forearm and wrist markers due to the impossibility of using active markers on the hand itself. Simply minimizing these errors does not result in optimal estimates. Rather, the errors shown in Figure 5 can be utilized to estimate unmeasured hand movements. To investigate this further, we compared the ball–hand error as a function of time during the hold phase when the ball is held in the hand (Figure 8). This analysis revealed systematic deviations between the ball and our estimate of hand position, with lateral displacement most extreme at the time of the throw and vertical displacements at the time of both catch and throw. While the reason for these discrepancies is most likely due to the method of estimating hand position, which due to limitations in motion capture not being able to track the hand, was a simple extrapolation, so these deviations, in turn, enable us to estimate the actual hand position with wrist movements. The V (and LV) methods likely offer the most veridical picture of vertical hand movement, but it is not obviously clear which offers the best reconstruction of the lateral movement. We reconstructed using the V and LV methods to determine which method could capture more realistic movements based on estimated wrist movement. In natural juggling, the wrist is difficult to adduct at the catch position. As throwing balls requires more active wrist movement than catching, we focused on catch points and regarded adducted write movement as an unrealistic movement. Figure 9 shows the extrapolated hand position estimate (without wrist movement) and the reconstructed ball trajectory for the V and LV methods. The V method underestimated the lateral position of the catch location, resulting in the cycle-by-cycle lateral mismatch between the ball and hand at the time of the catch. The lateral mismatch of the trajectory reconstructed by the V method could be interpreted as a large ulnar (towards the midline, with palm facing up) deviation of the wrist of the performer catching a ball, but the magnitude of mismatch is not physically possible. Figure 3, which showed a different subject from Figure 9, also revealed the underestimation of lateral movement. Considering the unrealistic depiction of the V method, the LV method is clearly the best choice for these data.

### 4.3. Assumptions

The models were designed under several assumptions for simplicity that might affect the accuracy. First, we assumed the ball position extracted from the video was accurate. However, we have potentially unaccounted for error in ball position due to hand occlusion. In a situation where a minor error is acceptable, such as a soccer game, occlusion can be detected to estimate an occluded object [20]. However, juggling is challenging because it involves important events that make a sudden change in ball position, necessitating differentiating from hand occlusion that could result in the same change. At the same time, occlusion is likely to be associated with the events. This issue was not relevant to our analysis because we excluded frames around the throw and catch events where occlusion might be most important. Second, we could not directly measure hand position in this present study and estimated it by extending a line along the forearm and wrist markers, neglecting the wrist bending movements. From our results, this assumption appeared to be cut both ways. As shown in Ind, coregistration could result in the wrong trajectory because of a lack of data on wrist movement. Meanwhile, the other methods reflected information about wrist movements even though they were not measured directly. We could take advantage of the gravitational constraint to reinterpret errors in a ball-to-hand position recovered via the V method as wrist-bending movements, enabling us to exploit it as a pseudo-reference. Another aspect associated with our assumptions is the possibility of relative movement between the body and camera and nonlinearities in the camera coordinates. The effect of relative movement between the body and camera is readily estimated by looking at variations in the angle between the camera plane and the coronal body plane and variations in translational displacements. In this particular dataset, these were negligible, thus justifying their exclusion, but they could readily be incorporated into the models in other situations in which there was more movement of the body relative to the camera. Camera spatial nonlinearity was neglected as the camera lens was of medium focal length, but for use with other lenses, or accuracy at the edges of the frame, calibration would be advised. For future studies, we could take a video image of a large grid placed at the location of the juggler to allow us to test the video image’s spatial linearity. If it is non-linear, we should first apply a nonlinear warping of the image and then use our linear methods on the warped ball coordinates. On geometric principles, more camera angles can improve localization; indeed, that is the principle of all current marker-based systems, which use large arrays of cameras. The reconstruction methods presented in our study could easily be extended to the case of multiple video cameras. In this present paper, we successfully demonstrated how adding a single additional commodity video camera to an existing multi-camera system can greatly extend the power of motion capture to a range of situations involving bodies and free-flying objects.

### 4.4. Importance of Timing

Finally, uncertainty about the throw and catch times also should be considered because those times were used to segment the data into hold and flight phases, which were used differently in coregistration. We evaded this potential trouble by eliminating three frames at both ends of each phase. This conservative way might unnecessarily lose useful parts of the information. We inserted the throw and catch times manually, watching all frames on video. Alternative methods that have been used in the past are inserted events based on ball kinematics, such as changes in vertical acceleration [12]. In our case, errors on the order of ±1 frame for both catch and throw (~17 ms for a 60 Hz video frame rate) could represent at most about 6% of the hold period, which averaged approximately 520 ms across participants (or ±2 frames out of 32 frames). While that is negligible in terms of time, an inspection of Figure 8 shows that the deviation between ball and hand was the greatest around the catch and throw, at the extreme of about 3 cm on average, suggesting that a greater error would be observed if we did not remove the six frames in total for each phase. To study the effect of such errors, one could conduct a permutation analysis by randomly jittering the throw and catch points by up to ±1 frame and investigate the effects on our analysis. In our case, we conducted a maximal bounds worst-case analysis without discarding any frames, comparing our results to the two extreme edges of the error space. We discovered increased errors averaged across subjects in the extended phase. The difference in errors between the extended phase and shortened phase was 1.40, 1.62, 1.49, and 1.53 [cm] for the Ind, L, V, and LV models, respectively. Given that errors resulting from our methods were approximately 3 cm, this 1.5 cm should not be ignored. Again, we evaded this issue by discarding enough samples around the events to make sure to obtain samples only from a correct phase, and so the possibility of mixing samples in a different phase by the uncertainty about event times is not directly relevant to our analysis. We confirmed that the uncertainty about event time should be considered because it may involve wrong ball kinematics that should be observed in a different phase.

## 5. Conclusions

In this present study, we demonstrated methods that were able to successfully coregister free-flying thrown and caught objects in video with marker-based motion capture. The best method (the LV model) utilized a combination of physical constraints during flight and ball holding as well as hand motion constraints to result in a coregistration that allowed us to accurately quantify ball flight and with the additional benefit of enabling the accurate recovery of unmeasured wrist movements. The methods (L and V) that partially employed the availability of the constraints and applied homogeneous scaling across dimensions were suboptimal. The methods developed here, which combine the fitting of constraints and reverse reconstruction of unobserved variables could be broadly applicable to other studies of movement dynamics in which not all objects can be instrumented with active markers. A number of general lessons can be drawn from this work. First, simple least squares fitting is not the best approach because the object/body ‘error’ will often, in fact, contain important behavioral information. Considering the least square fitting is likely to be exploited in general, defining task constraints should be prioritized. From this work, we developed several recommendations for future experimental studies of complex object/body interactions that can be difficult to study using marker-based motion capture alone and for which currently available markerless motion capture is not accurate enough. Such cases can benefit from a hybrid approach with the addition of video capture to marker-based systems. The recommendations are as follows: (1) identify task constraints (e.g., in the case of juggling, intervals for free flight of an object in a task that can be matched with the known gravitational acceleration and intervals of physical interaction between a performer and an object); (2) design a method to maximize the use of constraints, such as the LV model in our case, considering the importance of scaling different dimensions using different constraints; (3) avoid simple error minimization as fitting errors may contain behaviorally relevant information; (4) examine and extract meaningful information from the errors.

## Data Availability

The data presented in this study are available on request from the corresponding author.
